# Randomized Controlled Trial of a Volitional Help Sheet to Encourage Weight Loss in the Middle East

**DOI:** 10.1007/s11121-017-0807-z

**Published:** 2017-06-23

**Authors:** Christopher J. Armitage, Soud Alganem, Paul Norman

**Affiliations:** 1 Manchester Centre for Health Psychology, Division of Psychology and Mental Health, School of Health Sciences, Manchester Academic Health Science Centre, Faculty of Biology, Medicine and Health, University of Manchester, Coupland Street, Oxford Road, Manchester, M13 9PL UK; 2 Kuwait University, Kuwait City, Kuwait; 3 University of Sheffield, Sheffield, UK

**Keywords:** Brief intervention, Volitional help sheet, Health behavior change, Transtheoretical model, Implementation intentions, Overweight

## Abstract

In the Middle East, the prevalence of overweight/obesity is 80%; however, no studies have yet tested the efficacy of interventions to promote weight loss. The aim of the present study was to test the ability of implementation intentions formed using a volitional help sheet to support weight loss among people who were overweight/obese. Participants (*N* = 216) enrolling in a weight loss program in Kuwait were randomly allocated either to form implementation intentions using a volitional help sheet (intervention group) or to use the volitional help sheet to think about critical situations and appropriate responses but not form implementation intentions (control group). The main outcome measure was weight at 6-month follow-up. Participants in the intervention condition lost significantly more weight (6.15 kg; −6.58% initial body weight) than those in the control condition (3.66 kg; −4.04% initial body weight), *M*_diff_ = 2.55% initial body weight, *SE*_diff_ = .92, *t*(214) = 2.76, *p* = .006, 95%CI = .73, 4.36, *d* = .38. The present study is the first to show that implementation intentions work beyond a Western context and that the volitional help sheet could be used in a variety of cultural contexts to enhance weight-loss programs.

**Electronic supplementary material:**

The online version of this article (doi:10.1007/s11121-017-0807-z) contains supplementary material, which is available to authorized users.

In 2008, more than 1.4 billion adults were overweight (>500 million obese) worldwide and the prevalence of adult obesity had doubled since 1980; the prevalence of overweight/obesity continues to rise (World Health Organisation 2013). Most of the research into tackling the overweight/obesity problem has been conducted in the USA and Europe, yet the volume of research does not reflect the distribution of excess weight globally. The present research was conducted in Kuwait, where 80% of adults are overweight (Al Rashdan and Al Nesef 2010; cf. 38% in England, see Eastwood 2011). To date, no studies have tested the efficacy of interventions to promote weight loss anywhere in the Middle East meaning that the applicability of Western approaches to weight loss are open to question. The present study was designed to test a very brief, theory-based psychological intervention—a volitional help sheet—to augment weight loss among people who were overweight/obese enrolled in a commercial weight loss program.

## Implementation Intentions

The basis for the present intervention was Gollwitzer’s (1993) concept of implementation intentions, which have been shown to be effective in translating people’s motivation into action (Gollwitzer and Sheeran 2006). People form implementation intentions by linking in memory a critical situation with an appropriate response. For example, one possible critical situation could be “if I am tempted to eat when I am depressed (or down)” that could be linked to the appropriate response “then I will do something else instead of eating when I need to relax or deal with tension.” Laboratory studies show that when critical situations are encountered, appropriate responses are triggered automatically (Gollwitzer and Sheeran 2006). Among studies of implementation intention-based interventions to promote weight loss, effect sizes have ranged between *d* = .13 (Luszczynska et al. 2007) and *d* = .66 (Armitage et al. 2014).

## Volitional Help Sheets

Typical applications of implementation intention-based interventions in non-laboratory settings ask participants to generate their own implementation intentions without support (e.g., Armitage 2009) or with the support of health professionals (e.g., Luszczynska et al. 2007). However, participants may find it difficult to generate good quality plans by themselves and the cost of using health professionals to support participants to make plans may be prohibitive. Volitional help sheets are one means of overcoming these potential problems because they are designed to provide a standard tool with which people can form their own implementation intentions (Armitage 2008). The volitional help sheet provides participants with a list of critical situations they may encounter and the responses they might find useful to help them change their behavior. Specifically, the volitional help sheet draws on the transtheoretical model of change (Prochaska and DiClemente 1983) by operationalizing critical situations as temptations (i.e., situations in which health-risk behaviors might be triggered) and appropriate responses as “processes of change,” which are ten strategies by which health-protecting behavior is sustained or health-risk behavior is changed (e.g., *counter conditioning*, namely, finding a substitute for the problem behavior, as in the example above).

The volitional help sheet has been tested in randomized controlled studies in several domains including smoking (Armitage 2008, 2016), alcohol consumption (Armitage and Arden 2012), self-harm (Armitage et al. 2016), dietary intake (Armitage 2015), and physical activity (Armitage and Arden 2010), but only once in the domain of weight loss. Armitage et al. (2014) randomly allocated 72 overweight participants who were participating in a commercial weight-loss program in the UK to either an intervention (volitional help sheet) condition or a control (distracter task) condition. Participants in both conditions lost significant amounts of weight at 1-month follow-up, but those in the intervention condition lost significantly more than those in the control condition (*d* = .66). Armitage et al.’s (2014) study is the most exacting test of the volitional help sheet to date because it demonstrated impact over and above the effects of an ongoing commercial weight-loss program.

Despite the promising findings associated with Armitage et al.’s (2014) application of the volitional help sheet to weight loss, there were two notable limitations. First, the Armitage et al. (2014) study—and all volitional help sheet studies to date, with the exception of one conducted in Malaysia (see Armitage et al. 2016)—was conducted in the UK. Given that the issue of weight loss is pertinent to the Middle East, it would be valuable both to tackle a critical public health problem and test the volitional help sheet in a new cultural context. Second, the follow-up period was restricted to 1 month, which means the longer-term effects on weight loss were not assessed. It would be valuable to see whether the volitional help sheet could affect weight loss up to 6 months post-intervention, the time at which health behavior changes have been argued by Prochaska and DiClemente (1983) to be “maintained.” Indeed, the US National Heart, Lung and Blood Institute (2005) define successful weight loss as reducing body weight by 10% from baseline in 6 months, and it is important to establish whether the volitional help sheet can support clinically significant change.

The aims of the study were to see whether the volitional help sheet could lead to significant weight loss over and above the effects of a behavior change program in the Middle East. It is hypothesized that relative to the control condition, the volitional help sheet will boost weight loss at 6-month follow-up.

## Method

### Participants

The sample was recruited as they were enrolling in a commercial weight-loss program in Kuwait. All people enrolling for the first time on the program who were overweight or obese (i.e., body mass index ≥25) were eligible to participate. The program cost approximately $8 per week and consisted of weekly one-to-one advice or group sessions from trained consultants around eating plans, goal setting, and image therapy. Participants were free to leave the program at any point and all participants in the study received the same commercial weight-loss program throughout the study period.

At baseline, clinic staff who were blind with respect to condition, measured the height and weight of all people enrolling in the program. All people who were overweight (i.e., BMI ≥25) were invited by receptionists to participate in a study that was “aiming to aid weight management” and completed baseline questionnaires. Informed consent was obtained before the study began and participants were assured of their confidentiality and anonymity, and of their right to withdraw from the study or have their data removed at any point. The appropriate internal review board gave approval to conduct the research. Although permission was given to approach people enrolling for the first time in the program, the company that ran the program did not formally endorse the intervention and no incentive was offered to participants.

### Design

The study design was a randomized controlled trial with two parallel conditions (intervention versus control) and pre-post-test (baseline and 6-month follow-up). Six-month weight was the main outcome measure.

### Intervention

All participants were presented with the volitional help sheet on a single occasion (see Appendix; Armitage et al. 2014), which was appended to the end of baseline questionnaires (not reported here). The volitional help sheet was presented as a table with 12 rows and two columns each containing lists of critical situations and appropriate responses. The weight efficacy lifestyle questionnaire (Clark et al. 1991) was used to source critical situations; measures of transtheoretical model processes of change (Cancer Prevention Center 2011) were used to source appropriate responses. The temptation items were translated into “if” statements, for example: “if I am tempted to eat when I am depressed (or down)”; the processes of change items were translated into “then” statements, for example, “then I will do something else instead of eating when I need to relax or deal with tension” (see Chapman et al. 2009). Each statement had a tick-box next to it.

In addition to receiving the volitional help sheet, participants in both control and intervention conditions were informed that detecting tempting situations and recognizing ways to overcome those temptations was effective in aiding weight loss. Participants in the intervention condition were asked to *draw links* between as many critical situations and appropriate responses as they wanted (see Armitage et al. 2014), thereby forming implementation intentions by linking in memory critical situations with appropriate responses (Gollwitzer 1993). In contrast, participants in the control condition were simply asked to *tick* all the critical situations and appropriate responses that applied to them personally, but were not asked to link critical situations with appropriate responses and therefore did not form implementation intentions.

### Measures

All materials were translated into Arabic and then back-translated into English. In addition to demographic information, participants were asked how many times in the preceding year they had seriously (i.e., for at least 1 week) tried to lose weight. At 6-month follow-up, weight (kg) was taken from participants’ clinic records by clinic staff who were blind to condition.

### Procedure

**Fig. 1:**
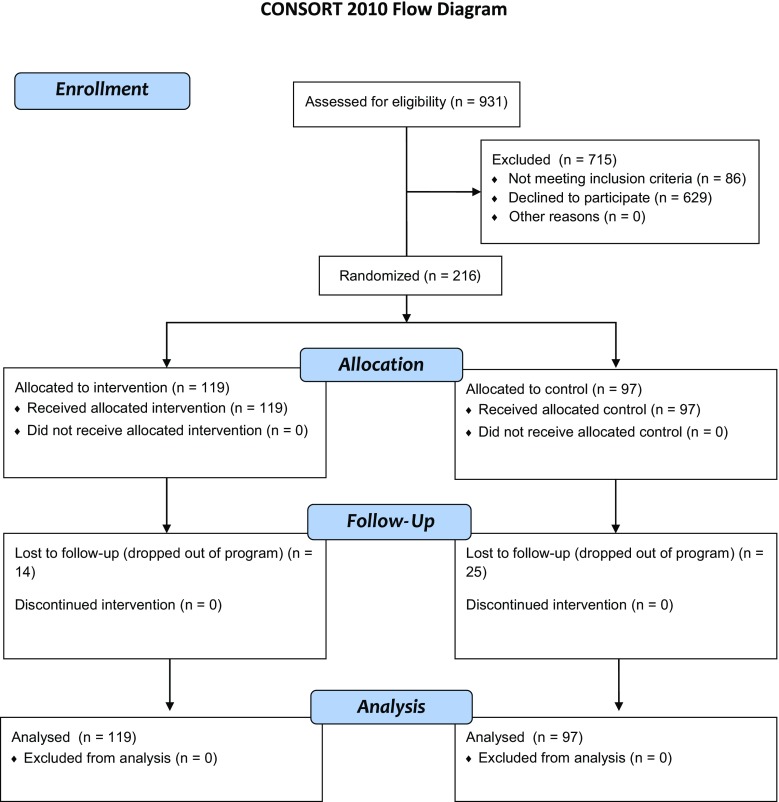
CONSORT 2010 Flow Diagram

Potential participants were approached at the time they enrolled in the weight-loss program and were invited to participate in a study that was “aiming to aid weight management” (Fig. 1). The volitional help sheets were placed at the end of identical-looking questionnaires. Questionnaires with the intervention/control instructions were sorted into random order using coin tosses by the researcher prior to data collection (i.e., the allocation ratio was 1:1 with no restrictions). Receptionists then distributed these identical-looking questionnaires and participants were left alone to complete the baseline questionnaire in the clinic. Once the baseline questionnaire was completed, the participant returned it to the receptionist in a sealed opaque envelope. Participants were then weighed by clinic staff who were blinded to condition and the receptionist made a note on the participant’s file to retrieve follow-up weight measurements. Thus, the people who weighed participants at baseline and follow-up were blind with respect to condition.

### Statistical Analysis

The commercial weight-loss company offered us a 12-month window in which to collect the data (January–December 2010) and so the decision to stop recruiting was pragmatic. Observed power was 0.79 (*α* = .05). The effect of the intervention on weight was tested using a mixed-measures ANOVA with *condition* (intervention versus control) as the between-participants independent variable and *time* (baseline versus 6-month follow-up) as the within-participants independent variable. The US National Heart, Lung and Blood Institute (2005) define successful weight loss as reducing body weight by 10% in 6 months. Chi-square analysis was used to see if the numbers of participants reducing their body weight by 10% or more in the 6-month follow-up period differed significantly between conditions. The reported analyses used intention-to-treat (baseline observation carried forward). Per-protocol analyses were also conducted.

## Results

### Sample Characteristics

Baseline characteristics of the sample are presented in Table 1. The sample consisted of 127 women and 61 men (*n* = 28 declined to report gender) aged *M* = 30.11 years (*SD* = 11.20). All participants were overweight with a body mass index (BMI) ≥25 and baseline weight ranged between 49.2 and 173.0 kg (*M* = 89.48, *SD* = 18.14). Weight at 6-month follow-up was assessed from records; however, 39 participants dropped out of the program (14 from the intervention group, 25 from the control group) and did not provide follow-up weight (Fig. 1). Attrition analysis revealed few significant differences between those who remained in the study and those who dropped out in terms of the measured baseline characteristics. However, significantly more people dropped out of the control group than the intervention group, *χ* ^2^(1, *N* = 216) = 7.09, *p* = .01, and those who dropped out were taller, *t*(214) = 2.02, *p* = .05, than those who remained in the study. Thus, data were analyzed according to intention to treat, with baseline observations carried forward. Per-protocol analyses (i.e., with those not lost to follow-up) made no substantive difference to the reported findings.

**Table 1:** Baseline characteristics of the sample

Variables	Full sample, *N* = 216		Intervention, *n* = 119		Control, *n* = 97	
	*M*	*SD*	*M*	*SD*	*M*	*SD*
Age (years)	30.11	11.20	30.94	10.31	29.42	11.99
BMI	33.92	5.87	33.11	5.63	34.91	6.03
Height (meters)	1.62	0.08	1.62	0.08	1.62	0.09
Weight (kg)	89.48	18.14	87.49	17.54	91.92	18.65
Number of serious weight-loss attempts	14.27	32.25	10.62	27.32	18.03	36.42
	*N*	%	*n*	%	*n*	%
Gender						
Women	127	58.80	63	52.94	64	65.98
Men	61	28.24	40	33.61	21	21.65
Missing	28	12.96	16	13.45	12	12.37

### Effects of the Volitional Help Sheet

There was a significant main effect of condition on weight, *F*(1, 214) = 5.81, *p* = .017, *η*_p_^2^ = .03, *d* = .35, and a main effect of time, *F*(1, 214) = 102.46, *p* < .001, *η*_p_^2^ = .32, *d* = 1.37, with participants losing weight over time (Table 2). Consistent with predictions, there was a significant condition *x* time interaction, *F*(1, 214) = 5.34, *p* = .022, *η*_p_^2^ = .02, *d* = .29. Over the course of the 6-month study, participants in the intervention group lost *M* = 6.15 kg (*M* = 6.58%, *SE* = .68) of their initial body weight compared with participants in the control group who lost *M* = 3.86 kg (*M* = 4.04%, *SE* = .62) of their initial body weight, *F*(1, 214) = 7.64, *p* = .006, *η*_p_^2^ = .03. Thus, the volitional help sheet boosted the effectiveness of the weight loss program by 38.60%, *M*_diff_ = 2.55%, *SE*_diff_ = 0.92, *t*(214) = 2.76, *p* = .006, 95%CI = .73, 4.36, *d* = .38.

**Table 2:** Effects of the volitional help sheet on weight between baseline and follow-up

Variables	Intervention, *n* = 119		Control, *n* = 97	
	*M*	*SD*	*M*	*SD*
Baseline weight (kg)	87.49	17.55	91.92	18.65
6-month follow-up weight (kg)	81.34	15.28	88.06	17.84

Mean values are raw scores and unadjusted for baseline weight or BMI. There was a significant condition *x* time interaction, *F*(1, 214) = 5.34, *p* = .022, *η*_p_^2^ = .02, *d* = .29.

In the present study, 27/119 (22.69%) participants in the experimental condition reduced their body weight by 10% or more in the 6-month follow-up period, compared with 14/97 (14.43%) in the control group (i.e., “successfully” lost weight, see US National Heart, Lung and Blood Institute 2005). The effect of the intervention on the numbers of people achieving successful weight loss (i.e., losing ≥10% body weight between baseline and 6-month follow-up) approached conventional statistical significance compared with participants in the control condition, *χ* ^2^(1, *N* = 216) = 2.37, *p* = .062, *d* = .21.

## Discussion

### Summary

This is the first study to test the efficacy of a weight-loss intervention in the Middle East, a region in which overweight/obesity is highly prevalent (e.g., Al Rashdan and Al Nesef 2010). It is also the first evidence to suggest that the previous promising findings associated with the volitional help sheet to bring about behavior change over a 6-month period. The key finding was that the volitional help sheet was effective in significantly augmenting weight loss over and above the effects of an ongoing commercial weight management program. The following discussion considers the practical and theoretical implications of the findings.

### Weight Loss and the Volitional Help Sheet

Despite the fact that the intervention and control groups completed very similar tasks and were exposed to identical and numerous additional behavior change techniques, the instruction to use the volitional help sheet to form implementation intentions (Gollwitzer 1993) was effective in reducing weight in an overweight sample in a field setting. The present study therefore replicates the findings of Armitage et al. (2014) in a new cultural context and extends them by showing that the effects can be sustained over a 6-month period. In order to minimize the demand characteristics placed on both the participants and the commercial weight-loss program, the intervention was deployed on a minimal basis by incorporating it into a paper-and-pencil questionnaire. This means that the volitional help sheet could easily be embedded into routine practice as an additional tool to encourage weight loss.

Even though the volitional help sheet was deployed with a “light touch,” more people in the intervention group (*n* = 27/119) lost 10% of their initial body weight than in the control group (*n* = 14/97) providing evidence to suggest that the volitional help sheet could have clinically significant effects (US National Heart, Lung and Blood Institute 2005). Moreover, given the trivial cost to implement the intervention, the volitional help sheet could be routinely incorporated in weight-loss programs. In addition, the low cost and high potential public health reach of the volitional help sheet could also make it applicable to help people outside formal programs to lose weight. In future research, it would be valuable to explore the kinds of behavior change techniques (e.g., goal setting), providers (e.g., clinicians, psychologists) and technologies (e.g., online) that may augment the effects of the volitional help sheet for changing behavior.

Theoretically, the findings confirm that simply being aware of critical situations and appropriate responses is not sufficient to bring about change: Linking critical situations with appropriate responses is the “active” ingredient of implementation intentions (e.g., Gollwitzer and Sheeran 2006). Given that weight at 6-month follow-up was assessed from participants’ clinic records, we were unable to assess potential mediators of the effect of the intervention. Although considerable laboratory research shows that enhanced cue accessibility and automation of cue-response links is key (e.g., Gollwitzer and Sheeran 2006), these are not yet amenable to measurement in the field. However, emerging research suggests that the ability of implementation intentions to enhance the accessibility of cues and to automate cue-response links might be manifest in changing people’s habits (e.g., Armitage 2016) and boosting capacity for self-regulation (e.g., Armitage 2015, 2017). Further research is required to identify the mechanisms that explain the operation of implementation intentions that are amenable to measurement in the field.

### Cross-Cultural Work

The fact that we were able to show that the volitional help sheet was adaptable to a Middle Eastern population implies that many of the techniques employed in Western weight management programs may well translate into the Middle East. The present research therefore makes a small step towards expanding the reach of behavior change research, which clearly needs to expand its sights beyond the West (see also Armitage et al. 2016).

### Limitations

It is important to acknowledge some potential limitations of the study when considering the current findings. First, although we were able to demonstrate significant effects on weight loss, clearly it would be valuable to establish the longer-term effects. Nevertheless, the present findings show promise, and other implementation intention-based interventions have demonstrated significant effects up to 2 years post-baseline (e.g., Conner and Higgins 2010). Second, although our retention rate once people had been randomized into the study was very good, there was substantial reluctance to participate in the research in the first place, as only 23.20% of people who were approached initially accepted the invitation. Further research is needed to help understand why the participation rate was so low, but it is notable that we did not offer any material incentive for participation. Third, all the participants were in treatment, meaning they were highly motivated to lose weight: In order to meet the public health challenge posed by obesity, it would be valuable to explore the efficacy of the volitional help sheet outside of commercial weight-loss programs. Fourth, we did not publish a protocol or data analysis plan prior to data collection beginning and so the findings should be interpreted accordingly. Fifth, a simple randomization method was employed for pragmatic reasons that could have been open to bias. However, the questionnaires given to the intervention and control conditions were designed to look identical, including both groups being exposed to the volitional help sheet albeit with different instructions, thereby reducing the likelihood of randomization bias.

### Conclusion

The present study showed that the volitional help sheet produced clinically significant effects on weight loss among people enrolled in a weight management program in a Middle Eastern context. The volitional help sheet represents a very brief, low-cost, intervention that could be used to supplement ongoing weight-loss programs.

### Electronic supplementary material

ESM 1 (DOC 216 kb)

## Appendix

### Volitional Help Sheet for Weight Loss

We want you to plan to lose weight. Research shows that if people can spot situations in which they will be tempted to eat and then link them with a way to overcome those situations, they are much more likely to be successful in losing weight. On the left hand side of the page below is a list of common situations in which people feel tempted to eat; on the right hand side of the page is a list of possible solutions. For each situation that applies to you personally (left hand side), please draw a line linking it to a solution (right hand side) that you think might work for you. Please draw a line linking one situation to one solution at a time, but make as many (or as few) situation-solution links as you like.

## References

[CR1] Al Rashdan I, Al Nesef Y (2010). Prevalence of overweight, obesity, and metabolic syndrome among adult Kuwaitis: Results from community-based national survey. Angiology.

[CR2] Armitage CJ (2008). A volitional help sheet to encourage smoking cessation: A randomized exploratory trial. Health Psychology.

[CR3] Armitage CJ (2009). Effectiveness of experimenter-provided and self-generated implementation intentions to reduce alcohol consumption in a sample of the general population: A randomized exploratory trial. Health Psychology.

[CR4] Armitage CJ (2015). Field experiment of a very brief worksite intervention to improve nutrition among health care workers. Journal of Behavioral Medicine.

[CR5] Armitage CJ (2016). Evidence that implementation intentions can overcome the effects of smoking habits. Health Psychology.

[CR6] Armitage CJ (2017). Unpublished raw data.

[CR7] Armitage CJ, Arden MA (2010). A volitional help sheet to increase physical activity in people with low socioeconomic status: A randomized exploratory trial. Psychology and Health.

[CR8] Armitage CJ, Arden MA (2012). A volitional help sheet to reduce alcohol consumption in the general population: A field experiment. Prevention Science.

[CR9] Armitage CJ, Norman P, Noor M, Alganem S, Arden MA (2014). Evidence that a very brief psychological intervention boosts weight loss in a weight loss program. Behavior Therapy.

[CR10] Armitage CJ, Rahim WA, Rowe R, O'Connor RC (2016). An exploratory randomised trial of a simple, brief psychological intervention to reduce subsequent suicidal ideation and behaviour in patients admitted to hospital for self-harm. British Journal of Psychiatry.

[CR11] Cancer Prevention Center. (2011). *Weight control: Processes of change*. Retrieved from: http://www.uri.edu/research/cprc/measures/wght_ctrl_processes_change.html

[CR12] Chapman J, Armitage CJ, Norman P (2009). Comparing implementation intention interventions in relation to young adults’ intake of fruit and vegetables. Psychology and Health.

[CR13] Clark MM, Abrams DB, Niaura RS, Eaton CA, Rossi JS (1991). Self-efficacy in weight management. Journal of Consulting and Clinical Psychology.

[CR14] Conner M, Higgins AR (2010). Long-term effects of implementation intentions on prevention of smoking uptake among adolescents: A cluster randomized controlled trial. Health Psychology.

[CR15] Eastwood P (2011). Statistics on obesity, physical activity and diet: England, 2011.

[CR16] Gollwitzer PM (1993). Goal achievement: The role of intentions. European Review of Social Psychology.

[CR17] Gollwitzer PM, Sheeran P (2006). Implementation intentions and goal achievement: A meta-analysis of effects and processes. Advances in Experimental Social Psychology.

[CR18] Luszczynska A, Sobczyk A, Abraham C (2007). Planning to lose weight: Randomized controlled trial of an implementation intention prompt to enhance weight reduction among overweight and obese women. Health Psychology.

[CR19] Prochaska JO, DiClemente CC (1983). Stages and processes of self-change in smoking: Toward an integrative model of change. Journal of Consulting and Clinical Psychology.

[CR20] US National Heart, Lung and Blood Institute (2005) *Guidelines on overweight and obesity: Electronic textbook.* Retrieved from: http://www.nhlbi.nih.gov/guidelines/obesity/e_txtbk/txgd/4311.htm

[CR21] World Health Organisation (2013). Obesity and overweight. *WHO fact sheet*, *311*. Retrieved from: http://www.who.int/mediacentre/factsheets/fs311/en/

